# An Atomistic Study of the Tensile Deformation of Carbon Nanotube–Polymethylmethacrylate Composites

**DOI:** 10.3390/polym15132956

**Published:** 2023-07-05

**Authors:** Anshu Raj, Sk Md Ahnaf Akif Alvi, Khayrul Islam, Mohammad Motalab, Shuozhi Xu

**Affiliations:** 1 School of Aerospace and Mechanical Engineering, University of Oklahoma, Norman, OK 73019, USA; 2 Department of Materials Science and Engineering, Texas A & M University, College Station, TX 77843, USA; 3 Department of Mechanical Engineering and Mechanics, Lehigh University, Bethlehem, PA 18015, USA; 4 Department of Mechanical Engineering, Bangladesh University of Engineering and Technology, Dhaka 1000, Bangladesh

**Keywords:** molecular dynamics, carbon nanotube, polymethylmethacrylate, polymer, composite

## Abstract

There has been growing interest in polymer/carbon nanotube (CNT) composites due to an exceptional enhancement in mechanical, structural, thermal, and electronic properties resulting from a small percentage of CNTs. However, the performance of these composites is influenced by the type of polymer used. PMMA is a polymer of particular interest among many other polymers because of its biomaterial applications due to its biocompatibility, non-toxicity, and non-biodegradability. In this research, we utilized a reactive force field to conduct molecular dynamics simulations to investigate changes in the mechanical properties of single-walled carbon nanotube (SWCNT)-reinforced Poly (methyl methacrylate) (PMMA) matrix composites. To explore the potential of SWCNT-reinforced PMMA composites in these applications, we conducted simulations with varying CNT diameters (0.542–1.08 nm), CNT volume fractions (8.1–16.5%), and temperatures (100 K–700 K). We also analyzed the dependence of Young’s modulus and interaction energy with different CNT diameters, along with changes in fracture toughness with varying temperatures. Our findings suggest that incorporating a small amount of SWCNT into the PMMA polymer matrix could significantly enhance the mechanical properties of the resulting composite. It is also found that the double-walled carbon nanotube has roughly twice the tensile strength of SWCNT, while maintaining the same simulation cell dimensions.

## 1. Introduction

Carbon has many allotropic forms. Each allotropic form of carbon demonstrates many interesting properties. For example, in sp^2^ hybridization, elemental carbon can take the shape of numerous impressive structures [1], such as graphite (3D), graphene (2D), carbon nanotubes (1D), and fullerene (0D). In addition to the well-known graphite, Kroto et al. [2] discovered a carbon allotrope that could construct open and closed cages with a honeycomb atomic arrangement called Fullerene C60. In 1991, Iijima et al. [3] conducted an initial study of another carbon allotrope called carbon nanotubes (CNTs). Early findings involved nearby shell-separated multi-walled carbon nanotubes (MWCNTs). After two years, Iijima and Ichihashi [4] and Bethune et al. [5] discovered single-walled carbon nanotubes (SWCNTs). Among different allotropes of carbon, CNTs have received much attention in scientific research due to their exceptional mechanical, structural, thermal, and electrical properties having high strength, rigidity, and durability [6]. CNTs have been identified as prospective candidates for the next generation of reinforcing agents in composite materials. Many studies have concluded that adding CNTs in significantly lower amounts than typical microfibers drastically enhanced the mechanical characteristics of polymeric resin [7,8,9]. Mechanical characteristics also depend on the specific polymer selected and the number and caliber of CNTs included in the composite. Factors like number of atoms, structure, geometry, defects, and interfaces have a significant role that affect how well a material performs [10]. The CNT has gained interest as a possible reinforcement for structural and multifunctional composites [11].

The characteristics of CNT-reinforced polymer matrix composites have been measured experimentally, and the results have shown significant increases in the composite modulus over the matrix modulus. Ajayan et al. [12] published one of the earliest investigations into polymer/CNT composites, where they used mechanical mixing to distribute MWCNTs within a liquid epoxy resin randomly. Since then, plenty of other researchers have focused on the idea that adding CNTs might enhance polymer composites’ mechanical and electrical properties. Compared to the matrix value with 5% weight CNTs, Schadler et al. [13] discovered a 40% increase in the effective stiffness of CNT-reinforced epoxy. Qian et al. [8] discovered an increase in the effective modulus of CNT-reinforced polystyrene of 40% just by adding a 1% CNT. For MWCNT/epoxy resin composites, Xu et al. [14] also confirmed similar outcomes. Milo et al. [15] implanted CNTs in polyvinyl alcohol, whereas Peigney et al. [16] created composites with CNTs embedded in ceramic powders. The degree of mechanical reinforcement obtained may rely on several variables, including the degree of CNT alignment and the strength of the link between the polymer and the CNTs [17]. Khan et al. [18] enhanced the mechanical properties of polymer/nanodiamond composites via the surface optimization of detonation nanodiamonds.

Alongside experiments, computational modeling can offer some critical insights because it is challenging to regulate and test many of these features experimentally. To investigate polymer/CNT composites, theoretical and computational approaches have been used extensively. Molecular dynamics (MDs) simulations have successfully predicted the elastic characteristics of CNT–polymer composites. Frankland et al. [19] employed MDs simulations to depict the stress–strain behavior of polyethylene–CNT composites in both longitudinal and transverse directions. Mahboob et al. [20] employed MDs to examine the impact of changing four adjacent six-membered rings into two five-membered rings and two seven-membered rings on the mechanical characteristics of composites supplemented with SWCNTs. By utilizing MDs modeling, Tahreen and Masud [21] were able to accurately measure Young’s modulus, the modulus of elasticity, the modulus of rigidity, and the compressibility of polyethylene (PE) reinforced with SWCNTs. Using experimental data, Kamal et al. [22] investigated the mechanical behavior of carbon fiber–amine functionalized MWCNT/epoxy composites and determined Young’s modulus and Poisson’s ratio. Subsequently, Sharma et al. [23] analyzed the interfacial properties of MWCNT/epoxy composites that had been functionalized with an amine. The compass force field [24] is well suited for applications in condensed phases because it employs a hybrid methodology that combines ab initio and empirical techniques. However, it cannot accurately reflect the formation and dissolution of chemical bonds. The compass is limited to predicting stress–strain curves within the harmonic region. A force field, such as the reactive force field (ReaxFF) [25], can be used to get around this restriction.

ReaxFF is a multibody potential that employs a bond-order-based approach, resulting in a seamless energy transition between different molecules. Xiong et al. [26] developed a model for a CNT-reinforced PE composite, incorporating an interfacial covalent-bonded interaction. To achieve this, they used ReaxFF, which is designed especially for hydrocarbons, and were able to accurately replicate the experimentally observed Young modulus of 1000 GPa for a CNT. Using MDs simulations, Zaminpayma [27] investigated the interactions between CNTs and polythiophene (PT), PE, and poly(p-phenylenevinylene) (PPV), and used ReaxFF to analyze the impact of diameter, temperature, and polymer type on interaction energy. Islam et al. [28] also investigated the mechanical properties of a CNT–polyoxymethylene (POM) composite using ReaxFF.

Among many different polymers, a specific synthetic polymer generated from the methyl methacrylate monomer is known as PMMA, also known as poly [1-(methoxycarbonyl)-1-methyl ethylene] from a hydrocarbon viewpoint and poly (methyl 2-methyl propanoate) from an ester perspective [29]. PMMA is a widely used polymer of the polyacrylate class. It is non-biodegradable and biocompatible. In order to attain biodegradable qualities, it is utilized either by itself or as a matrix or component phase in various products [30]. Apart from its mechanical strength and stability, low cost, and easy manufacturability, it holds promise for biomedical use due to its non-toxicity and minimal inflammatory reactions with tissues [31]. Due to the presence of neighboring methyl groups (CH3), the polymer structure of PMMA is unable to tightly pack in a crystalline manner and spin freely around the C-C bonds, indicating its amorphous thermoplastic nature [31]. As such, PMMA exhibits a variety of applications, including but not limited to optical, sensors, pneumatic actuation, conductive devices, and analytical separation [32,33,34,35]. PMMA is also beneficial in dental filling [36,37], implants [38,39], microspheres [30,40], microcapsules [41,42], bone grafting [43,44], contact lenses [45,46], polymer electrolytes, polymer viscosity, and drug delivery via electro-diffusion or electro-osmotic flow [47,48,49,50,51]. PMMA is highly versatile due to a range of advantageous properties that it possesses, including transparency, resistance to environmental factors, impact resistance, low moisture absorption, and biocompatibility [52]. These attributes make it suitable for use in a variety of applications. PMMA exhibits high mechanical strength, with a high Young’s modulus and low elongation at breakage. This rigidity prevents it from breaking upon rupture, making it one of the most durable and scratch-resistant thermoplastics [53,54].

In recent decades, researchers have conducted extensive studies on the behavior of PMMA mixed with CNTs. PMMA with CNTs or other inorganic components is essential in nanotechnology due to its compatibility and ease of processing as a polymer moiety. Wang et al. [55] employed poly (styrene-co-acrylonitrile) with poly (methyl methacrylate)-g-MWCNTs to synthesize CNT–polymer composites. Skountzos et al. [56] and Rahmat et al. [57] elucidated the equilibrium structure and dynamic behavior of PMMA chains in an interaction with a CNT via MDs. Malagù et al. [58] studied the effects of size and chirality effect on glass transition temperature and ordering in CNT-PMMA. Wang et al. [59] developed a multiscale approach to analyze the non-linear vibration behavior of PMMA/CNT composite plates. As previously mentioned, CNT-PMMA composites exhibit distinct properties, but the effects of varying parameters such as CNT diameter, CNT volume percentage, and working temperature on their mechanical properties have yet to be fully explored. Our work aims to bridge this gap and shed light on the behavior of PMMA/CNT composites under different conditions.

The present study employs ReaxFF with a charge calculation approach that takes into account the dependence on molecular geometry, enabling the accurate evaluation of polarization phenomena and redistribution of partial atomic charges in response to the structural transformation of molecular species or clusters [25,60]. By bridging the gap between quantum chemical and empirical force fields, this force field achieves a favorable balance between the accuracy and the computational cost. ReaxFF has been utilized by numerous researchers to forecast interactions between various hydrocarbons [61] as well as polymer–CNT composites [62,63]. It is for this reason that we have opted to employ this particular force field to model the CNT-PMMA composite in our current study.

This paper explores the mechanical properties of CNT-reinforced PMMA composites by utilizing ReaxFF to examine the impact of interfacial covalent-bonded interactions. Additionally, we studied how CNT diameter, volume percentage, and temperature affected interaction energy. Finally, we calculated the stress–strain curves of the CNT-PMMA composite to determine its Young’s modulus, ultimate tensile strength, ultimate tensile strain, and fracture toughness. Our investigation has revealed that the aforementioned parameters have a significant impact on the mechanical characteristics.

## 2. Materials and Method

### 2.1. Force Field (ReaxFF)

To investigate the interaction between CNT and a polymer chain, we utilized the Large-Scale Atomic/Molecular Massively Parallel Simulator (LAMMPS, version 3 March 2020), a free MDs simulator [64]. Our investigation employed the ReaxFF force field, which takes both covalent and non-covalent interactions into account and is dependent on bond ordering [65]. It is a force field that employs several concepts to determine bond order and includes both covalent and non-covalent interactions. It utilizes universal bond distance–bond order and bond order–bond energy relationships that facilitate the proper dissociation of bonds from individual atoms. The force field incorporates terms like angle and torsion (shown in Equation (1)) that are smoothly reduced to zero when bonds are broken.

Moreover, ReaxFF provides Coulomb and Morse (van der Waals) potentials to characterize all atoms’ non-bonded interactions. ReaxFF parameters were generated from quantum chemistry simulations, which involved the dissociation of bonds and reactions of small molecules, as well as the geometry data and heat of formation of various stable hydrocarbon compounds. The ReaxFF approach has proven to be effective in describing these data, owing to its capacity to seamlessly integrate quantum chemical and empirical force-field-based computational techniques. This feature allows for a more comprehensive understanding of the underlying chemical processes, thereby enabling accurate predictions of system behavior. This enables ReaxFF to simulate the systems that consist of thousands of atoms [25].

In general, ReaxFF adds up different types of partial energy contributions to determine the total energy of the system [25], which is

*E*_system_ = *E*_bond_ + *E*_over_ + *E*_under_ + *E*_val_ + *E*_pen_ + *E*_tors_ + *E*_conj_ + *E*_vdWalls_ + *E*_coulomb_ (1)

where these contributions consist of bond energy (*E*_bond_), the bond energy of over- and undercoordinated atoms (*E*_over_ and *E*_under_), valence angle energy (*E*_val_), angle penalty energy of over- and undercoordinated atoms (*E*_pen_), torsion angle energy (*E*_tors_), the contribution of conjugation effects to molecular energy (*E*_conj_), and Coulomb interaction energy (*E*_coulomb_).

In our model, there was no *E*_pen_ energy contribution because no atom shares two double bonds. However, even after fixing the initial bond order, some over-coordination may have still existed in the system, which was addressed by the penalty energy of *E*_over_. In addition, *E*_under_ was introduced to consider the coordination effect caused by resonance. The total energy of the system takes into account all of the energy contributions, including the parameters used in the above formulae and the specific values of hydrocarbon parameters employed in our recent MDs simulation [25].

We conducted a validation of ReaxFF by analyzing a CNT with an armchair (6,6) configuration, which had a length of 4.92 nm and a diameter of 0.815 nm. ReaxFF was used to characterize the interactions between carbons. The structure was then minimized and deformed to measure stress and strain, and from which, Young’s modulus was calculated.

The derived CNT results agree with the experimental results, with minor exceptions. These minor differences can be attributed to using CNTs of varying diameters and inevitable flaws in the experimental data, as reported by Kamal et al. [66], whereby CNT flaws dramatically reduce the CNT modulus. Salvetat et al. [67] used an atomic force microscope on two ends of clamped nanotubes, while Krishnan et al. [68] employed the thermal vibration of CNTs. However, Qiang et al. [69] and WenXing et al. [70] employed MDs to forecast armchair CNTs with changing lengths and diameters. All of these results are pretty similar to those produced by our simulation. So, the result shows the reliability and precision of ReaxFF (Table 1).

### 2.2. Molecular Model and MDs Simulation

**Figure 1:**
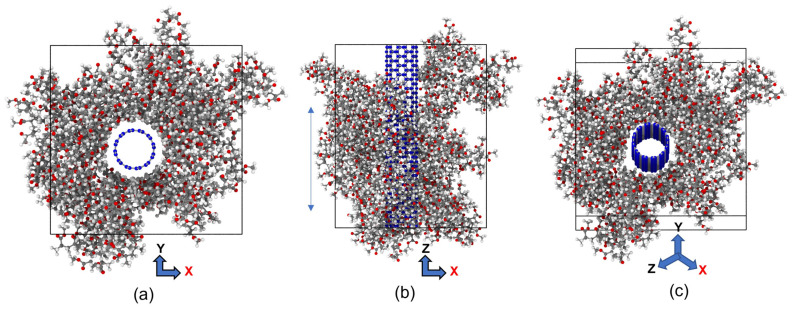
The MD model of CNT-PMMA nanocomposite, (**a**) front view, (**b**) side view, and (**c**) lateral views (CNT carbon: blue; hydrogen: white; oxygen: red and PMMA carbon: grey).

Initially, we utilized BIOVIA Materials Studio [71] to create a single chain of polymethylmethacrylate and CNT crystal. Then, we packed the polymer chain around the SWCNT to form the unit cell of the PMMA/CNT composite used in our investigation, as shown in Figure 1.

A 0.1 fs constant integration time step was employed in all of the simulations. Then, the energy of the system was minimized using the conjugate gradient algorithm. To ensure appropriate temperature and zero pressure in all directions, we performed consecutive Nosé–Hoover-type thermostat (NVT) [72] and barostat (NPT) simulations for 0.5 ps each prior to applying the tensile stress.

We subjected the composite material to uniaxial deformation at a strain rate of 10^10^ s^−1^, while controlling the temperature variations under an NPT ensemble. This allowed us to create a stress–strain curve, and from which we determined the ultimate tensile strength, ultimate tensile strain, fracture toughness, and Young’s modulus of the material.

## 3. Results and Discussion

### 3.1. Effect of Diameters

In this study, we created a molecular structure of CNTs with three different diameters of 0.542, 0.815, and 1.08 nm, while keeping their length constant at 4.92 nm. These CNT models were then positioned at the center of a simulation cell with amorphous characteristics, featuring dimensions of 3 nm × 3 nm × 4.92 nm. A polymer chain consisting of 10 repeating units and 151 atoms was constructed and packed into the simulation through geometry optimization. The resulting nanocomposite structure had an initial density of 1.18 g/cm^3^ with 15, 13, and 10 chains randomly placed within the simulation cell.

We examined how a CNT and a PMMA chain interacted in the initial stage. When there is no covalent chemical bonding, electrostatic and van der Waals forces account for most of the interfacial bond strength in the molecular system. The strength of the bond between CNT and PMMA at the interface can be determined by measuring their interaction energy, Δ*E* [73], which is

Δ*E* = *E*_total_ − (*E*_CNT_ + *E*_PMMA_) (2)

In this method, the interaction energy between the CNT and PMMA can be determined by calculating the difference in the total potential energy of the system (*E*_total_), which includes both the polymer and CNT, and the potential energy of the CNT alone (*E*_CNT_), as well as the potential energy of the PMMA without CNT (*E*_PMMA_). Specifically, the interaction energy was obtained by measuring the difference between the minimum energy of the CNT-PMMA composite simulation cell and the energy at which the polymer matrix and the nanotubes are infinitely separated. Intense interaction energy indicates a robust adhesion between the polymer and CNT, determined by the interaction energy.

**Figure 2:**
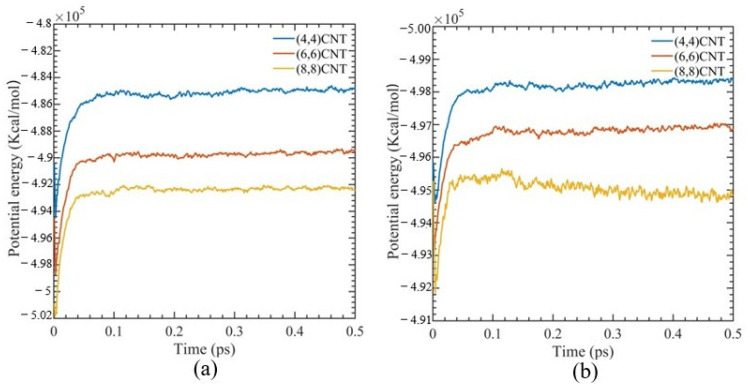
Potential energy evolution of CNT-PMMA composite for different CNT diameters using 0.5 ps of pressure equilibration at (**a**) 300 K and (**b**) 700 K.

Figure 2 illustrates the simulated box’s potential energy at various CNT diameters during NPT ensemble. The potential energy of the system shows minimal oscillation, and after 180 fs, the system fluctuations are less than 0.04%. This suggests that the system has equilibrated and has reached a stable structure without depending on the working temperature.

**Figure 3:**
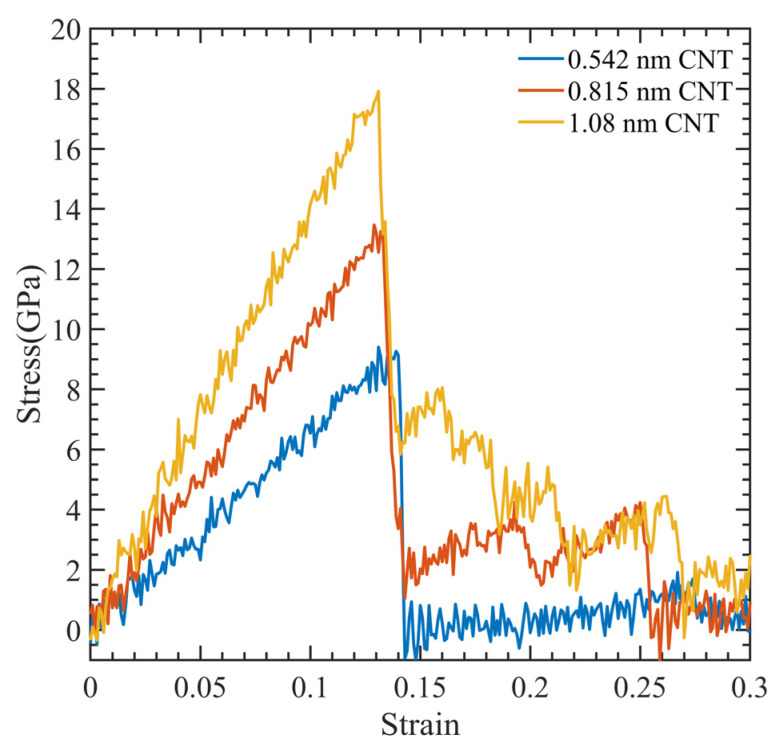
Stress-strain curve for CNT-PMMA composite at different CNT diameters.

**Figure 4:**
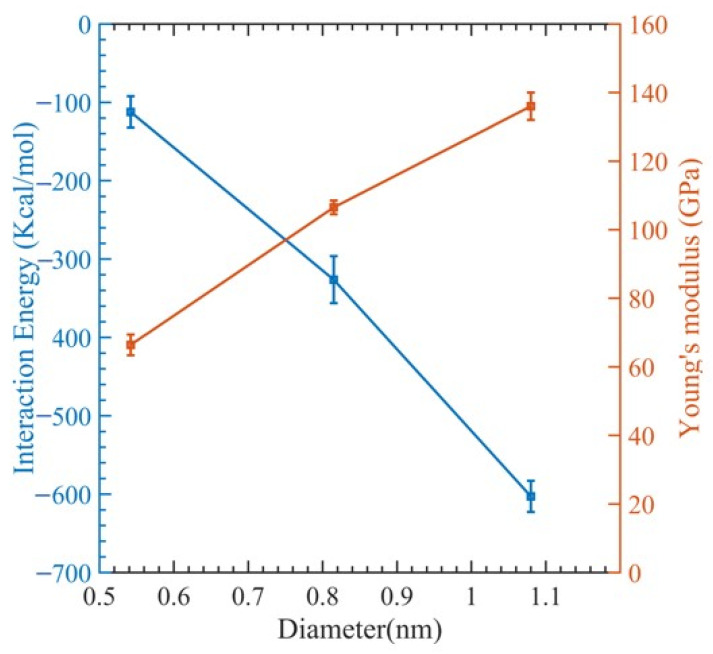
Change in interaction energy and Young’s modulus with respect to the diameter.

Figure 3 illustrates a stress–strain relationship, which enables the determination of ultimate tensile strength, ultimate tensile strain, and Young’s modulus. This relationship exhibits a brittle failure of the polymer matrix, and the final load-bearing capacity is due to the CNT fiber. Figure 4 displays the relationship between Young’s modulus and CNT radius. As the CNT diameter increases, an increase in Young’s modulus is observed, whereas the interaction energy of the system decreases. The relationship between the interaction energy and CNT diameter is determined by utilizing Equation (2).

**Figure 5:**
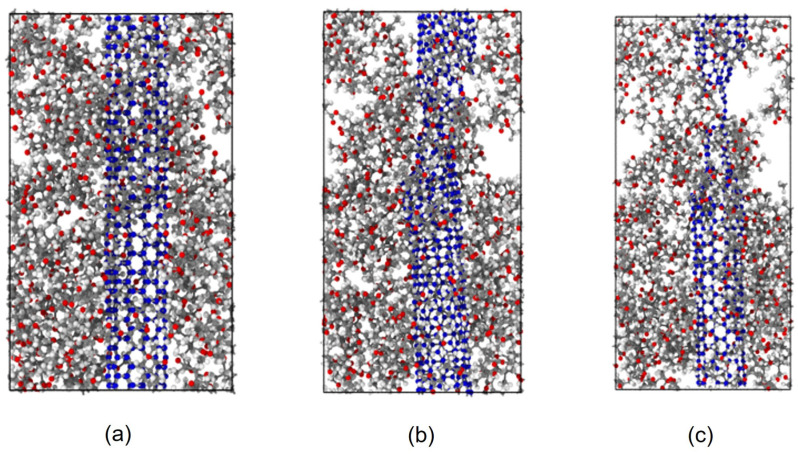
MD simulation snapshots at the atomic arrangement of (6,6) CNT-PMMA composite at different strain values, (**a**) strain = 0; (**b**) strain = 14%, and (**c**) strain = 26%.

In Figure 4, we can see how the interaction energy changes with varying CNT diameters. The range of interaction energy varies from −105 kcal/mol to −649 kcal/mol as the CNT diameter changes. This can be explained by observing that as the diameters of the CNTs rise, so does the surface contact area between them and the polymer matrix, which raises the interaction energy. However, there is a declining trend of interaction energy (Figure 4). This is because the polymer network undergoes significant distortion to make room for the CNTs. This implies that numerous small-diameter CNTs can be inserted instead of one large-diameter CNT, since they will increase the surface area while causing less polymer deformation [74]. Examining the fracture mechanism shown in Figure 5 shows that the polymer reinforcement is dragged away from the CNT reinforcement.

### 3.2. Effect of CNT Volume Fraction

The mechanical characteristics of the composite are significantly influenced by the CNT volume fraction [28,75]. We adopted a (6,6) SWCNT with a radius of 0.815 nm since the polymer matrix does not penetrate the CNTs and can be handled as a solid beam. To account for the contribution of the entire cross-section of the CNT, we included its effective volume fraction, denoted by ƒ_CNT_ as

(3) $${ƒ}_{CNT}=\frac{\pi {\left(R_{CNT}+\frac{h_{vdW}}{2}\right)}^{2}}{A_{cell}}$$

where A_cell_ is the unit cell’s cross-sectional area transverse to the nanotube axis and h_vdW_ is the equilibrium van der Waals separation distance between the CNT and the matrix. In this study, we determined the van der Waals separation distance, which is the distance between the CNT and PMMA due to their interfacial contact. The obtained value for this distance was 0.3035 nm.

Using Equation (3), we computed the CNT volume percentage, which was 16.5%, 11.7%, and 8.1%, depending on the cell dimension of the *x*- and *y*-axis, which was 2.5 × 2.5 × 4.92 nm, 3 × 3 × 4.92 nm, and 3.5 × 3.5 × 4.92 nm. In this study, a constant density of 1.18 g/cm^3^ was used to pack 9, 12, and 14 polymer chains into the simulation box mentioned above [59].

**Figure 6:**
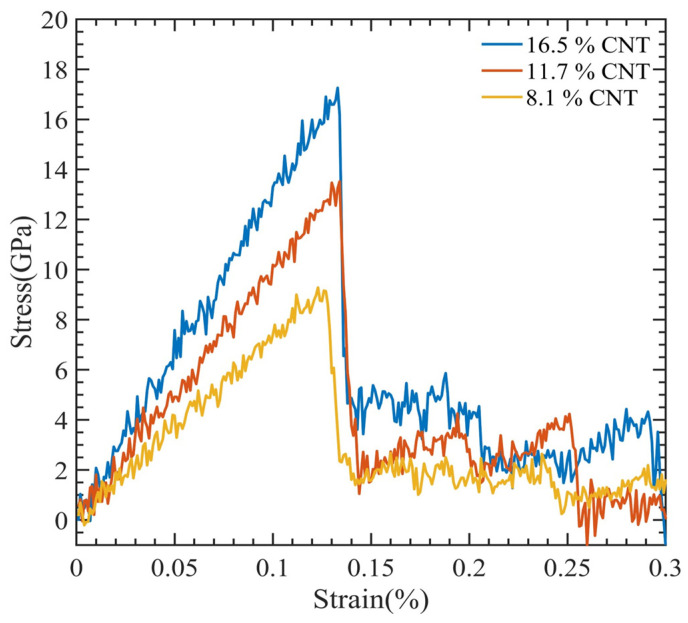
Stress-strain curve for CNT-PMMA composite at varying CNT contents.

**Figure 7:**
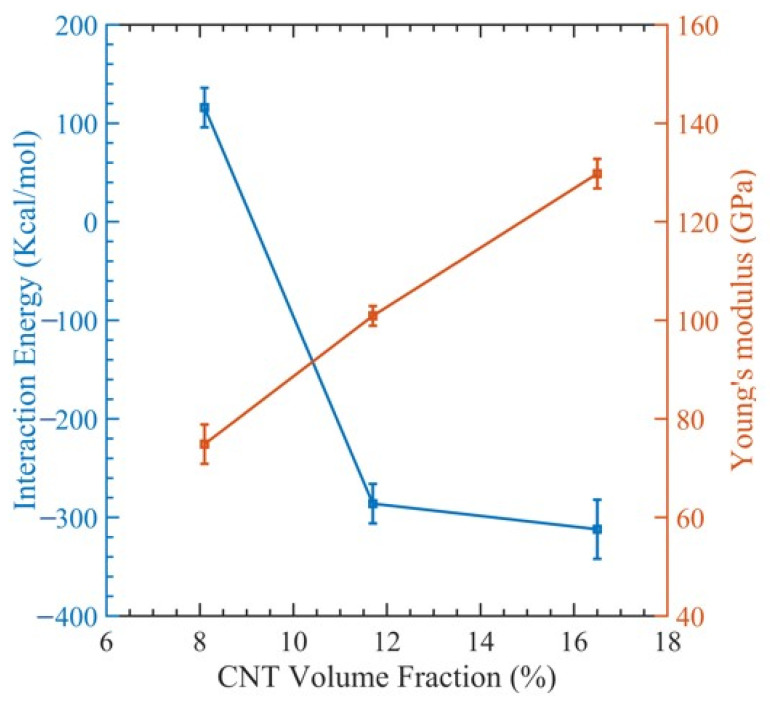
Change in interaction energy and Young’s modulus with respect to CNT contents.

The outcomes are shown as a stress–strain graph in Figure 6. After obtaining values from the previous section, we generated Figure 7 to visualize the relationship between Young’s modulus and the interaction energy with respect to the CNT content. This figure revealed an upward trend in the composite Young modulus with an increasing CNT volume fraction, which is very comparable to the finding made by Yue et al. [17] in their investigation of the poly{(m-phenylenevinylene)-co-[(2,5-dioctoxy-p-phenylene) vinylene]}–CNT composite through MDs simulation. As the volume fraction of the CNT increases, the interaction energy between the CNT and PMMA decreases, since less polymer chains have the opportunity to interact with the CNT. This can be observed in Table 2.

### 3.3. Effect of Temperature

**Figure 8:**
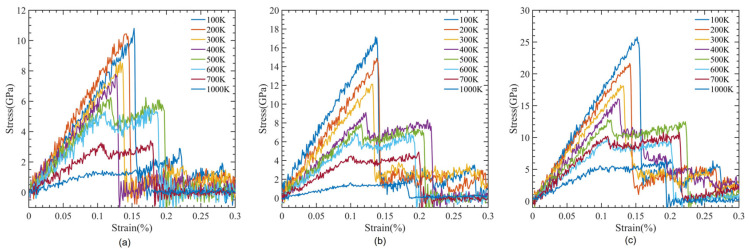
Stress-strain curve for CNT-PMMA composite at varying temperatures (**a**) 0.542 nm CNT, (**b**) 0.815 nm CNT, and (**c**) 1.08 nm CNT.

**Figure 9:**
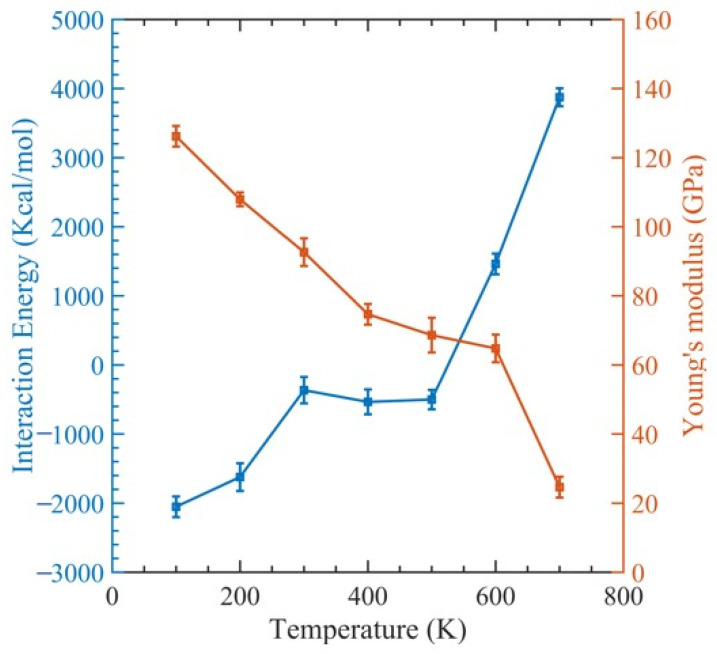
Change in interaction energy and Young’s modulus with respect to temperature.

**Figure 10:**
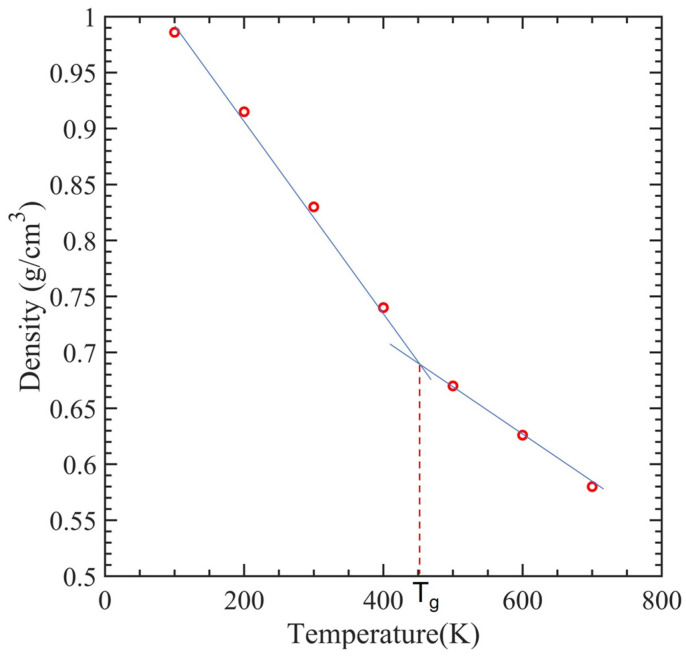
The recorded density of SWCNT-PMMA nanocomposite models as a function of temperature.

**Figure 11:**
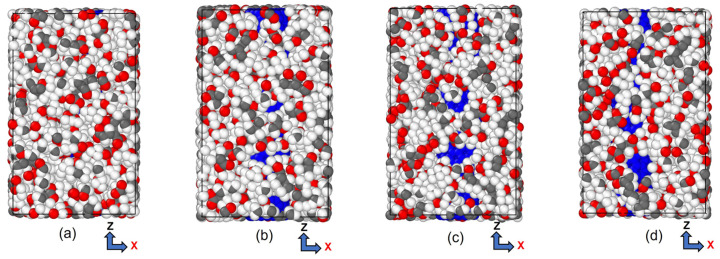
Visual representation of stable CNT-PMMA composite at (**a**) 100 K, (**b**) 400 K, (**c**) 500 K, and (**d**) 700 K.

CNTs with a diameter of 0.815 nm and a constant length of 4.92 nm were used to study the impact of temperature variation. There were 12 polymer chains of PMMA packed into the same amorphous cell dimension used for the concept states. The simulation box was heated to one of the seven temperatures, 100 K, 200 K…, 700 K, to achieve equilibrium before tensile deformation was applied. The stress–strain relationship obtained from the MDs simulation data was used to determine the ultimate tensile strength, ultimate tensile strain, and Young’s modulus of the composite, as illustrated in Figure 8. In Figure 9, the relationship between interaction energy and Young’s modulus is depicted, and it shows that as the temperature increases, Young’s modulus increases, while the interaction energy decreases gradually, except for 400 and 500 K. Because the composite’s glass transition occurred in this range of temperatures between 400 and 500 K [59], where a glassy state transforms into a viscous state, interaction energy is drastically lowered at these temperatures. In our case, the glass transition temperature was 451 K, which can be seen in Figure 10, which is consistent with 468.8 K obtained from another MDs simulation of PMMA/CNT [59]. In the lower temperature range, both the polymer and nanocomposite exist in a glassy state, which transitions to a rubbery state as the temperature increases. This transition between the glassy and rubbery states is referred to as the glass transition temperature T_g_. Another notable occurrence occurs at 700 K temperature when there is a significantly high value of interaction energy between the CNTs and PMMA due to strong bonding between them. This can be seen in Figure 11, where there is a distinct difference between the stable configuration of the CNT-PMMA composite at 100 and 700 K. The CNT can be seen in the composite’s xz plane which occurs due to the less densely packed PMMA chains at 700 K.

**Figure 12:**
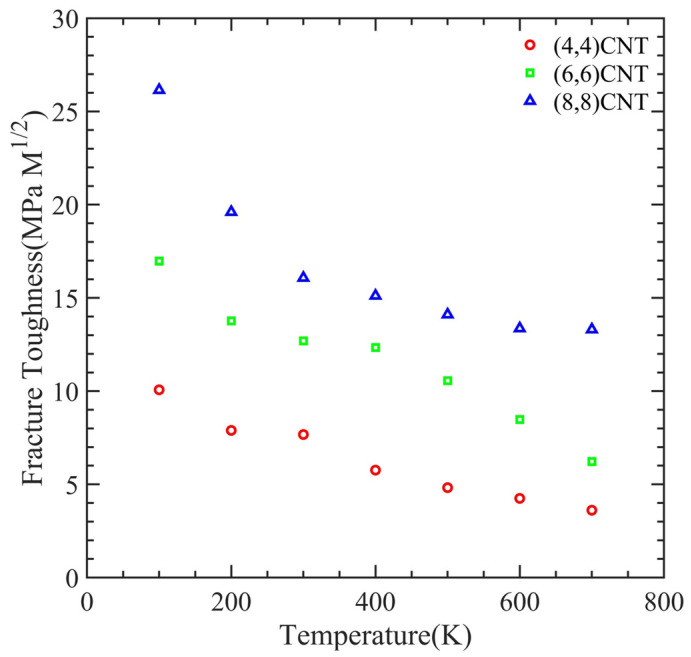
Variation of fracture toughness with the temperature at the varying orientation of CNT in PMMA-CNT composite.

Figure 12 illustrates the dependence of fracture toughness on simulation temperature while varying the orientation of the CNT in a PMMA-CNT composite. Fracture toughness refers to the energy per unit volume that a material can withstand before it fails. We evaluated this value by calculating the area stress–strain curve. Our results show that the fracture toughness decreases with an increase in simulation temperature. Moreover, we observed that a higher orientation of the CNT leads to higher fracture toughness.

### 3.4. Effect of Double-Walled CNT

Compared to the already robust SWCNTs, DWCNTs have been discovered to exhibit extraordinary strength properties. In this study, we created molecular models of CNTs with diameters of 0.542, 0.815, and 1.08 nm, while keeping their length constant at 4.92 nm. These CNT models were then positioned at the center of a simulation cell with amorphous characteristics, featuring dimensions of 3 × 3 × 4.92 nm.

**Figure 13:**
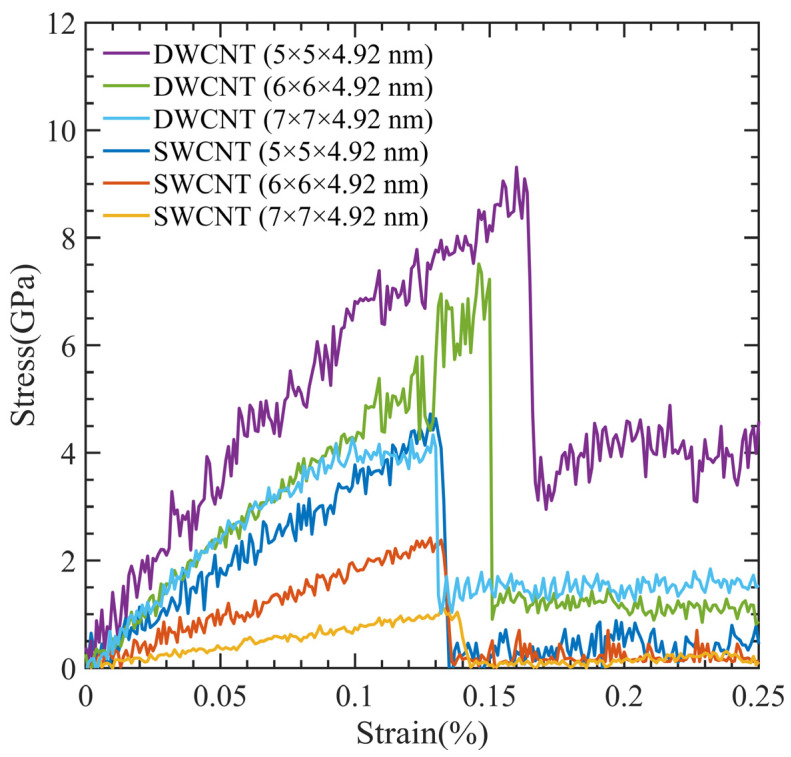
Comparison between SWCNT-PMMA and DWCNT-PMMA at different simulation cell dimensions.

Due to their exceptional characteristics, DWCNTs are commonly employed in applications that demand strong mechanical strength. Comparing the tensile strength of DWCNT (5 × 5 × 4.92 nm) and SWCNT (5 × 5 × 4.92 nm) models, Figure 13 shows that the DWCNT has double the tensile strength of the SWCNT. Apart from the number of CNT walls, these two models were essentially identical. The DWCNT models showed stronger strength and could resist higher strain percentages when we compared DWCNT (6 × 6 × 4.92 nm) and DWCNT (7 × 7 × 4.92 nm) models with their respective SWCNT models.

Our findings show that compared to SWCNTs, DWCNTs are stronger and can endure higher strain percentages. The design and development of sophisticated materials for a variety of industrial applications, such as aerospace, electronics, and medical devices, depends heavily on these findings.

## 4. Conclusions

In our study, we comprehensively examined the effects of CNT diameter, CNT volume fraction, temperature, and number of CNT walls on Young’s modulus, ultimate tensile strength, ultimate tensile strain, toughness, and interaction energy. To achieve this, we employed the MDs simulation method with ReaxFF potential. Our findings shed light on the complex relationships between these parameters and provide important insights into the behavior of CNT-PMMA composites. The following were revealed:

- Temperature is a significant factor affecting the mechanical properties of CNT-PMMA composites. As the temperature of the simulation rises from 100 K to 700 K, several key mechanical properties experience notable changes. Specifically, the ultimate tensile strength diminishes from 17.16 GPa to 4.90 GPa, the ultimate tensile strain decreases from 13.6% to 9.9%, and Young’s modulus undergoes a decrease from 126.16 GPa to 24.63 GPa.
- The diameter and volume fraction of CNTs are crucial parameters that determine the composite’s mechanical characteristics. It is observed that an increase in CNT diameter correlates with an increase in CNT volume fraction. This phenomenon, in turn, leads to notable enhancements in both the ultimate tensile strength, which rises from 9.40 GPa to 17.92 GPa, and Young’s modulus, which elevates from 66.41 GPa to 135.97 GPa. However, it is worth noting that the ultimate tensile strain is largely indifferent to the CNT size, remaining at approximately 13% throughout the experimental range.
- Our simulation results suggest that incorporating CNTs into PMMA can enhance the material’s mechanical properties, making it suitable for both low- and high-temperature applications.
- We observed that DWCNTs exhibit approximately twice the tensile strength of SWCNTs, while maintaining the same simulation cell dimensions.

## Figures and Tables

**Table 1:** Comparison of Young’s modulus of a CNT with previously published works.

Study	Young’s Modulus (GPa)
Our work	1130
Salvetat et al. (experimental) [67]	800 ± 410
WenXing et al. (LJ and REBO potential) [70]	935.805 ± 0.618
Qiang et al. (Morse potential) [69]	840 ± 20

**Table 2:** Comparison of ultimate tensile strength, ultimate tensile strain, and Young’s modulus of the material under different conditions, including variations in temperature, volume, and diameter.

Temperature (K)	CNT Diameter (nm)	CNT Volume Fraction (%)	Young’s Modulus (GPa)	Ultimate Tensile Strength (GPa)	Ultimate Tensile Strain (%)
300	0.542	7.5	66.41 ± 1.67	9.40	13.1
	0.815	11.7	106.52 ± 2.72	13.47	12.9
	1.08	16.3	135.97 ± 4.34	17.92	13.2
300	0.815	8.1	74.87 ± 4.12	9.28	12.3
		11.7	106.52 ± 2.72	13.52	13.4
		16.5	129.77 ± 2.96	17.26	13.3
100	0.815	11.7	126.16 ± 3.23	17.16	13.6
200			107.92 ± 2.01	14.89	13.8
300			92.65 ± 4.32	12.13	13.1
400			74.67 ± 3.11	9.11	12.2
500			68.79 ± 4.14	7.83	11.5
600			64.76 ± 3.87	6.93	10.7
700			24.63 ± 3.12	4.90	9.9

## Data Availability

The data that support the findings of this study are available from the corresponding author upon reasonable request.

## References

[1] Popov V.N. (2004). Carbon Nanotubes: Properties and Application. Mater. Sci. Eng. R Rep..

[2] Kroto H.W., Heath J.R., O’Brien S.C., Curl R.F., Smalley R.E. (1985). C60: Buckminsterfullerene. Nature.

[3] Iijima S. (1991). Helical Microtubules of Graphitic Carbon. Nature.

[4] Iijima S., Ichihashi T. (1993). Single-shell carbon nanotubes of 1-nm diameter. Nature.

[5] Bethune D.S., Kiang C.H., de Vries M.S., Gorman G., Savoy R., Vazquez J., Beyers R. (1993). Cobalt-catalysed growth of carbon nanotubes with single-atomic-layer walls. Nature.

[6] Shokrieh M.M., Rafiee R. (2010). Investigation of nanotube length effect on the reinforcement efficiency in carbon nanotube based composites. Compos. Struct..

[7] Kearns J.C., Shambaugh R.L. (2002). Polypropylene fibers reinforced with carbon nanotubes. J. Appl. Polym. Sci..

[8] Qian D., Dickey E.C., Andrews R., Rantell T. (2000). Load transfer and deformation mechanisms in carbon nanotube-polystyrene composites. Appl. Phys. Lett..

[9] Thostenson E.T., Chou T.-W. (2002). Aligned multi-walled carbon nanotube-reinforced composites: Processing and mechanical characterization. J. Phys. D Appl. Phys..

[10] Schaffer J.P., Saxena A., Antolovich S.D., Sanders T.H., Warner S.B. (1999). The Science and Design of Engineering Materials.

[11] Thostenson E.T., Ren Z., Chou T.-W. (2001). Advances in the science and technology of carbon nanotubes and their composites: A review. Compos. Sci. Technol..

[12] Ajayan P.M., Stephan O., Colliex C., Trauth D. (1994). Aligned Carbon Nanotube Arrays Formed by Cutting a Polymer Resin—Nanotube Composite. Science.

[13] Schadler L.S., Giannaris S.C., Ajayan P.M. (1998). Load transfer in carbon nanotube epoxy composites. Appl. Phys. Lett..

[14] Xu X., Thwe M.M. (2002). Mechanical properties and interfacial characteristics of carbon-nanotube-reinforced epoxy thin films. Appl. Phys. Lett..

[15] Du J., Cheng H.-M. (2012). The Fabrication, Properties, and Uses of Graphene/Polymer Composites. Macromol. Chem. Phys..

[16] Peigney A., Laurent C., Flahaut E., Rousset A. (2000). Carbon nanotubes in novel ceramic matrix nanocomposites. Ceram. Int..

[17] Han Y., Elliott J. (2007). Molecular dynamics simulations of the elastic properties of polymer/carbon nanotube composites. Comput. Mater. Sci..

[18] Khan M., Hamid A., Tiehu L., Zada A., Attique F., Ahmad N., Ullah A., Hayat A., Mahmood I., Hussain A. (2020). Surface optimization of detonation nanodiamonds for the enhanced mechanical properties of polymer/nanodiamond composites. Diam. Relat. Mater..

[19] Frankland S., Harik V., Odegard G., Brenner D., Gates T. (2003). The stress–strain behavior of polymer–nanotube composites from molecular dynamics simulation. Compos. Sci. Technol..

[20] Mahboob M., Islam M.Z. (2013). Molecular dynamics simulations of defective CNT-polyethylene composite systems. Comput. Mater. Sci..

[21] Tahreen N., Masud A. (2012). Investigation of the mechanical properties of polyethylene/carbon nanotube composite by molecular dynamics simulation. Int. J. Nano Biomater..

[22] Sharma K., Shukla M. (2014). Molecular modeling of the mechanical behavior of carbon fiber-amine functionalized multiwall carbon nanotube/epoxy composites. New Carbon Mater..

[23] Sharma K., Kaushalyayan K.S., Shukla M. (2015). Pull-out simulations of interfacial properties of amine functionalized multi-walled carbon nanotube epoxy composites. Comput. Mater. Sci..

[24] Sun H. (1998). COMPASS: An ab Initio Force-Field Optimized for Condensed-Phase ApplicationsOverview with Details on Alkane and Benzene Compounds. J. Phys. Chem. B.

[25] Van Duin A.C.T., Dasgupta S., Lorant F., Goddard W.A. (2001). ReaxFF: A Reactive Force Field for Hydrocarbons. J. Phys. Chem. A.

[26] Xiong Q.-L., Tian X.-G. (2015). Atomistic Modeling of Mechanical Characteristics of CNT-Polyethylene with Interfacial Covalent Interaction. J. Nanomater..

[27] Zaminpayma E. (2014). Molecular dynamics simulation of mechanical properties and interaction energy of polythiophene/polyethylene/poly(*p* -phenylenevinylene) and CNTs composites. Polym. Compos..

[28] Islam K., Saha S., Masud A.K.M. (2020). Molecular dynamics simulation of the mechanical properties of CNT-polyoxymethylene composite with a reactive forcefield. Mol. Simul..

[29] Stevens M.P. (1998). Polymer Chemistry: An Introduction.

[30] Yuksel N., Baykara M., Shirinzade H., Suzen S. (2011). Investigation of triacetin effect on indomethacin release from poly(methyl methacrylate) microspheres: Evaluation of interactions using FT-IR and NMR spectroscopies. Int. J. Pharm..

[31] Ali U., Karim K.J.B.A., Buang N.A. (2015). A Review of the Properties and Applications of Poly (Methyl Methacrylate) (PMMA). Polym. Rev..

[32] Hashim H., Adam N., Zaki N., Mahmud Z., Said C., Yahya M., Ali A. Natural Rubber-Grafted with 30% Poly(Methylmethacrylate) Characterization for Application in Lithium Polymer Battery. Proceedings of the 2010 International Conference on Science and Social Research (CSSR 2010).

[33] Shah J.J., Geist J., Locascio L.E., Gaitan M., Rao M.V., Vreeland W.N. (2006). Surface modification of poly(methyl methacrylate) for improved adsorption of wall coating polymers for microchip electrophoresis. Electrophoresis.

[34] Lee L.-H., Chen W.-C. (2001). High-Refractive-Index Thin Films Prepared from Trialkoxysilane-Capped Poly(methyl methacrylate)−Titania Materials. Chem. Mater..

[35] Adhikari B., Majumdar S. (2004). Polymers in Sensor Applications. Polym. Sci..

[36] Thomson L., Law F., James K., Matthew C., Rushton N. (1992). Biocompatibility of particulate polymethylmethacrylate bone cements: A comparative study in vitro and in vivo. Biomaterials.

[37] Kawaguchi T., Lassila L.V., Tokue A., Takahashi Y., Vallittu P.K. (2011). Influence of molecular weight of polymethyl(methacrylate) beads on the properties and structure of cross-linked denture base polymer. J. Mech. Behav. Biomed. Mater..

[38] Freitag C.P.F., Kruel C.R.P., Duarte M.E.S., Sanches P.R.E., Thomé P.R.O., Fornari F., Driemeier D., Teixeira F., Mollerke R.O., Callegari-Jacques S.M. (2008). Endoscopic implantation of polymethylmethacrylate augments the gastroesophageal antireflux barrier: A short-term study in a porcine model. Surg. Endosc..

[39] Itokawa H., Hiraide T., Moriya M., Fujimoto M., Nagashima G., Suzuki R., Fujimoto T. (2007). A 12 month in vivo study on the response of bone to a hydroxyapatite–polymethylmethacrylate cranioplasty composite. Biomaterials.

[40] Bux J., Manga M.S., Hunter T.N., Biggs S. (2016). Manufacture of poly(methyl methacrylate) microspheres using membrane emulsification. Philos. Trans. R. Soc. A Math. Phys. Eng. Sci..

[41] Pan X., Mercadé-Prieto R., York D., Preece J.A., Zhang Z. (2013). Structure and Mechanical Properties of Consumer-Friendly PMMA Microcapsules. Ind. Eng. Chem. Res..

[42] Kim J.-W., Lee K.-S., Ju H.-K., Ryu J.-H., Han S.-H., Chang I.-S., Kang H.-H., Oh S.-G., Suh K.-D. (2004). Microencapsulation of cholesteryl alkanoate by polymerization-induced phase separation and its association with drugs. J. Polym. Sci. Part A Polym. Chem..

[43] Nien Y.-H., Lin S.-W., Hsu Y.-N. (2013). Preparation and characterization of acrylic bone cement with high drug release. Mater. Sci. Eng. C.

[44] Sugino A., Miyazaki T., Kawachi G., Kikuta K., Ohtsuki C. (2007). Relationship between apatite-forming ability and mechanical properties of bioactive PMMA-based bone cement modified with calcium salts and alkoxysilane. J. Mater. Sci. Mater. Med..

[45] MacRae S.M., Matsuda M., Phillips D.S. (1994). The Long-term Effects of Polymethylmethacrylate Contact Lens Wear on the Corneal Endothelium. Ophthalmology.

[46] Hosaka S., Yamada A., Tanzawa H., Momose T., Magatani H., Nakajima A. (1980). Mechanical properties of the soft contact lens of poly(methyl methacrylate-N-vinylpyrrolidone). J. Biomed. Mater. Res..

[47] Isha A., Yusof N.A., Ahmad M., Suhendra D., Yunus W.M.Z.W., Zainal Z. (2006). A chemical sensor for trace V(V) ion determination based on fatty hydroxamic acid immobilized in polymethylmethacrylate. Sens. Actuators B Chem..

[48] Kost J., Langer R. (2001). Responsive polymeric delivery systems. Adv. Drug Deliv. Rev..

[49] Beruto D.T., Botter R., Fini M. (2001). The effect of water in inorganic microsponges of calcium phosphates on the porosity and permeability of composites made with polymethylmethacrylate. Biomaterials.

[50] Shi M., Kretlow J.D., Spicer P.P., Tabata Y., Demian N., Wong M.E., Kasper F.K., Mikos A.G. (2011). Antibiotic-releasing porous polymethylmethacrylate/gelatin/antibiotic constructs for craniofacial tissue engineering. J. Control. Release.

[51] Mishra S., Sen G. (2011). Microwave initiated synthesis of polymethylmethacrylate grafted guar (GG-g-PMMA), characterizations and applications. Int. J. Biol. Macromol..

[52] Heini P.F., Wälchli B., Berlemann U. (2000). Percutaneous transpedicular vertebroplasty with PMMA: Operative technique and early results. A Prospective Study for the Treatment of Osteoporotic Compression Fractures. Eur. Spine J..

[53] Harper C.A., Petrie E.M. (2003). Plastics Materials and Processes: A Concise Encyclopedia.

[54] van Krevelen D.W., Nijenhuis K.T. (2009). Properties of Polymers.

[55] Wang M., Pramoda K., Goh S.H. (2005). Enhancement of the mechanical properties of poly(styrene-co-acrylonitrile) with poly(methyl methacrylate)-grafted multiwalled carbon nanotubes. Polymer.

[56] Skountzos E.N., Mermigkis P.G., Mavrantzas V.G. (2018). Molecular Dynamics Study of an Atactic Poly(methyl methacrylate)–Carbon Nanotube Nanocomposite. J. Phys. Chem. B.

[57] Rahmat M., Hubert P. (2012). Molecular Dynamics Simulation of Single-Walled Carbon Nanotube—PMMA Interaction. J. Nano Res..

[58] Malagù M., Lyulin A., Benvenuti E., Simone A. (2016). A Molecular-Dynamics Study of Size and Chirality Effects on Glass-Transition Temperature and Ordering in Carbon Nanotube-Polymer Composites. Macromol. Theory Simul..

[59] Wang J.F., Yang J.P., Tam L.-H., Zhang W. (2021). Molecular Dynamics-Based Multiscale Nonlinear Vibrations of PMMA/CNT Composite Plates. Mech. Syst. Signal Process..

[60] Buehler M.J. (2008). Deformation and Dynamical Failure of Brittle Materials. Atomistic Modeling of Materials Failure.

[61] Liu F., Hu N., Ning H., Liu Y., Li Y., Wu L. (2015). Molecular dynamics simulation on interfacial mechanical properties of polymer nanocomposites with wrinkled graphene. Comput. Mater. Sci..

[62] Beese A.M., Sarkar S., Nair A., Naraghi M., An Z., Moravsky A., Loutfy R.O., Buehler M.J., Nguyen S.T., Espinosa H.D. (2013). Bio-Inspired Carbon Nanotube–Polymer Composite Yarns with Hydrogen Bond-Mediated Lateral Interactions. ACS Nano.

[63] Saha B., Furmanchuk A., Dzenis Y., Schatz G.C. (2015). Multi-step mechanism of carbonization in templated polyacrylonitrile derived fibers: ReaxFF model uncovers origins of graphite alignment. Carbon.

[64] AThompson A.P., Aktulga H.M., Berger R., Bolintineanu D.S., Brown W.M., Crozier P.S., Veld P.J.I., Kohlmeyer A., Moore S.G., Nguyen T.D. (2021). LAMMPS—A flexible simulation tool for particle-based materials modeling at the atomic, meso, and continuum scales. Comput. Phys. Commun..

[65] Plimpton S. (1995). Fast Parallel Algorithms for Short-Range Molecular Dynamics. J. Comput. Phys..

[66] Sharma K., Saxena K.K., Shukla M. (2012). Effect of Multiple Stone-Wales and Vacancy Defects on the Mechanical Behavior of Carbon Nanotubes Using Molecular Dynamics. Procedia Eng..

[67] Salvetat J.-P., Briggs G.A.D., Bonard J.-M., Bacsa R.R., Kulik A.J., Stöckli T., Burnham N.A., Forró L. (1999). Elastic and Shear Moduli of Single-Walled Carbon Nanotube Ropes. Phys. Rev. Lett..

[68] Krishnan A., Dujardin E., Ebbesen T.W., Yianilos P.N., Treacy M.M.J. (1998). Young’s Modulus of Single-Walled Nanotubes. Phys. Rev. B.

[69] Lu Q., Bhattacharya B. (2005). Effect of randomly occurring Stone–Wales defects on mechanical properties of carbon nanotubes using atomistic simulation. Nanotechnology.

[70] WenXing B., ChangChun Z., WanZhao C. (2004). Simulation of Young’s modulus of single-walled carbon nanotubes by molecular dynamics. Phys. B Condens. Matter.

[71] BIOVIA Materials Studio—BIOVIA—Dassault Systèmes. https://www.3ds.com/products-services/biovia/products/molecular-modeling-simulation/biovia-materials-studio/.

[72] Faiyad A., Munshi A.M., Islam M., Saha S. (2021). Deformation mechanisms of Inconel-718 at the nanoscale by molecular dynamics. Phys. Chem. Chem. Phys..

[73] Harik V. (2001). Ranges of applicability for the continuum beam model in the mechanics of carbon nanotubes and nanorods. Solid State Commun..

[74] Gissinger J.R., Pramanik C., Newcomb B., Kumar S., Heinz H. (2017). Nanoscale Structure–Property Relationships of Polyacrylonitrile/CNT Composites as a Function of Polymer Crystallinity and CNT Diameter. ACS Appl. Mater. Interfaces.

[75] Arash B., Park H.S., Rabczuk T. (2015). Mechanical properties of carbon nanotube reinforced polymer nanocomposites: A coarse-grained model. Compos. Part B Eng..

